# Magneto-Optical Transport Properties of Type-II Nodal Line Semimetals

**DOI:** 10.3390/ma14113035

**Published:** 2021-06-02

**Authors:** Yanmei Sun, Jing Li, Hui Zhao, Meimei Wu, Hui Pan

**Affiliations:** Department of Physics, Beihang University, Beijing 100191, China; lijing111@bua.edu.cn (J.L.); hzhao@buaa.edu.cn (H.Z.); 18811735028@163.com (M.W.); hpan@buaa.edu.cn (H.P.)

**Keywords:** magneto-optical response, landau levels, type-II nodal line semimetals

## Abstract

We investigate the magneto-optical transport properties and Landau levels of type-II nodal line semimetals. The tilted liner dispersion in type-II nodal line semimetals makes the conduction band and valence band asymmetric, and Landau levels are coupling in the presence of a magnetic field. We find the background of absorption peaks is curved. The oscillation peaks are tailless with the change of magnetic field. Through tuning tilt term, we find the absorption peaks of optical conductivity change from incomplete degenerate structure to splitting double peaks structure. We also find interband absorption peaks is no longer zero in the imaginary part of Hall conductivity. With the change of the tilt term, the contribution of the absorption peak has two forms, one is that the negative peak only appears at high frequencies, and the other is two adjacent peaks with opposite signs. In addition, the resistivity, circularly polarized light and magnetic oscillation of Hall conductivity are studied.

## 1. Introduction

Recently, as new type of topological semimetals, nodal line semimetals (NLSs) have attracted extensive attention due to their unique band structure and potential applications in nanoelectronics [1,2,3,4,5,6]. Unlike Dirac semimetals [7,8,9] and Weyl semimetals [2,10,11,12,13,14], where the valence and conduction bands intersect at discrete points in the Brillouin zone, the NLSs have extended energy band touching points, forming a protected one-dimensional line or closed loop [15,16,17,18,19]. Many exotic physical properties caused by this special topological structure have been studied, including magnetic susceptibility [20], Landau levels (LLs) quantization [21,22,23], quantum oscillations [24,25,26], and optical conductivities [27,28].

According to the band dispersion slope around the band crossing points, NLSs can be classified as type-I, type-II, and type-III [29,30,31]. For type-I NLS with opposite slope sign in conduction and valence bands, its magneto-optical conductivity have studied in Physica B: Condensed Matter, 2020, 599, 412478. In the type-II NLS, the nodal ring is formed by two bands which are tilted dispersion along the same direction. Compared with other type NLSs, there are significant differences in magnetic, transport and topological properties [30]. In recent years, the theoretical and experimental research on type-II NLSs have made some progress. The compound $K_{4}P_{3}$ was theoretically verified to be the first type-II NLS with a pair of type-II node loops [30]. $Mg_{3}Bi_{2}$ as a type-II NLS has been confirmed theoretically and experimentally [31,32]. A new type-II NLS model based on a two-band cubic lattice was pointed out, and it was discovered that the landau energy level collapsed under the influence of a magnetic field [33]. Based on first-principles calculations, the pure titanium and pure zirconium metal have also been confirmed to have type-II nodal line state [34,35]. Through these studies, the type-II nodal line phase has been realized in many materials, and the related topological characteristics have been studied. However, there are relatively few studies on the magneto-optical transport of type-II NLSs. In the presence of a uniform magnetic field, the electron continuous energy spectrum will be transformed into discrete Landau levels, and the optical transition between the Landau energy levels will produce magneto-optical conductivity resonance peaks. In experiments, these resonance peaks can reflect the basic band structure [36,37,38], and the magneto-optical properties as a response function are also a particular experimental measure for topological materials [39,40]. Recent studies on magneto-optical properties have provided very useful information for topological semimetals [41,42,43,44,45,46,47,48]. Therefore, this work based on Kubo formula to study the magneto-optical transport properties of type-II NLS is valuable. We find that the resonance peaks of the magneto-optical conductivity exhibit some unique characteristics. These features are very closely related to the tilted linear dispersion, which makes the conduction band and valence band asymmetric. When the magnetic field exists, the Landau levels are coupled and asymmetric. We find that these asymmetrical LLs make the transitions from −(n−1) to *n* and −n to (n−1) require different energy. This result that absorption peaks of longitudinal conductivity are incomplete degenerate structure. The incomplete degenerate structure change into splitting double peaks structure by tuning tilt term. We observe non-linear background for absorption peaks. However, the long tail vanishes when the chemical potential falls in the coupled LLs. Moreover, we find that intreband peaks are no longer zero in Hall conductivity. With the change of tilt term, the energies of the absorption peaks from that the negative are at higher frequency relative to positive change into two adjacent peaks with opposite signs. And the longitudinal resistivity tensor and Hall resistivity are calculated. We also study the effect of tilt term and chemical potential on absorption peak of circularly polarized light. Besides, the magnetic oscillation of Hall conductivity versus temperature and tilt terms are presented.

Our paper is organized as following. In Section 2, a low energy effective model of type-II NLS is introduced and the band structure is derived when the perpendicular magnetic field is applied to the plane of the ring. The expressions of longitudinal conductivity and Hall conductivity are calculated according to the Kubo formula. In Section 3, we provide numerical results for longitudinal conductivity, Hall conductivity, resistivity and circularly polarized lights. We also show the magnetic oscillation of Hall conductivity versus temperature and tilt terms. Finally, we give our conclusion and summarize our results in Section 4.

## 2. Model Hamiltonian

We use k·p model at Γ about k-quadratic terms, which is obtained by the low-energy model of type-II NLS [29] as follows:

(1) $$\begin{array}{c} H\left(p\right)=\left[\begin{array}{cc} p_{x}^{2}+p_{y}^{2} & ip_{z}\\ -ip_{z} & \Delta +\gamma (p_{x}^{2}+p_{y}^{2}) \end{array}\right]. \end{array}$$

**Figure 1:**
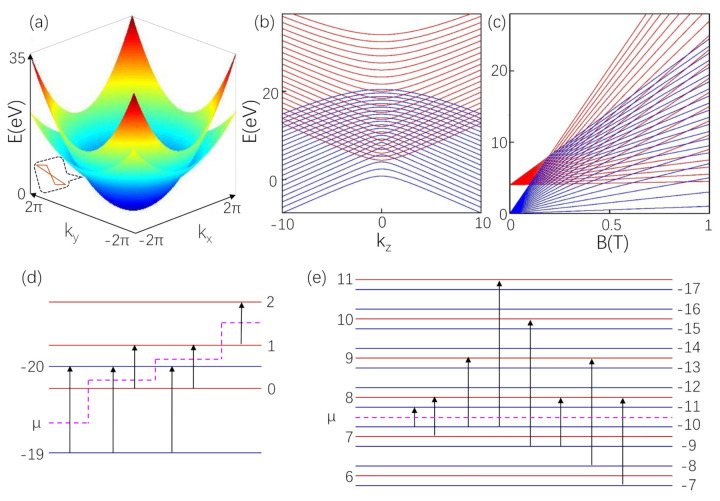
(**a**) The low energy dispersion of type-II NLS at $k_{z}=0$. Two cones with different slopes form type-II NLS. (**b**) is LLs structure along $k_{z}$ at B=0.8 T. (**c**) The LLs as a function of *B* at $k_{z}=0$ by Equation (4). Allowable optical transitions are indicated by black arrows. (**d**) Only intraband transition are marked in LLs for B=0.1 T. (**e**) is for B=0.8 T and μ=12.8 eV. The red (blue) LLs represent positive (negative) branch, denoted by n≥(≤)0 index. The dotted pink line represents the position of the chemical potential. The parameters N=20, γ=0.5, Δ=4 eV, and e=1.

The Δ is the gap. The γ(0≤γ<1) stands for the tilt of the valence band. The low energy dispersion in momentum space with $k{}_{z}=0$ is shown in Figure 1a. Two cones cross at $E=\frac{\Delta }{e(1-\gamma )}$ and form a ring. The linear dispersion of each point in nodal ring is tilted and the slope signs are the same. Considering magnetic filed B = $B\hat{z}$ in type-II NLS, the gauge is $A{}_{z}$ = 0, $A{}_{x}=-By/2$ and $A{}_{y}=Bx/2$.

By replacing the standard Peierls and the ladder operator representation [40,49], the annihilation $a=\frac{l_{B}}{\sqrt{2}}\left(k_{x}-ik_{y}\right)$ and creation operators $a^{†}=\frac{l_{B}}{\sqrt{2}}\left(k_{x}+ik_{y}\right)$ are used, where $l_{B}=1/\sqrt{eB}$, and $k_{x}=p_{x}+\frac{eBy}{2}$, $k_{y}=p_{y}-\frac{eBx}{2}$, $k_{z}=p_{z}$. The Hamiltonian of type-II NLS is written as ($\hbar =c=k_{B}=1$):

(2) $$H\left(k\right)=\left(\begin{array}{cc} \frac{1}{l_{B}^{2}}\left(a^{†}a+aa^{†}\right) & ik_{z}\\ -ik_{z} & \Delta +\frac{\gamma }{l_{B}^{2}}\left(a^{†}a+aa^{†}\right) \end{array}\right).$$

For solutions to eigenvalue HΨ=EΨ, taking

(3) $$\begin{array}{c} \Psi =\left(\begin{array}{c} A_{n}|n\rangle \\ B_{n}|n\rangle \end{array}\right), \end{array}$$

we gain

(4) $$\begin{array}{ccc} E_{n,\lambda ,k_{z}} & = & \frac{1}{2}\left[E_{B}\left(1+\gamma \right)+\Delta \right]\\ & + & \frac{1}{2}\lambda \sqrt{{\left[E_{B}\left(1+\gamma \right)+\Delta \right]}^{2}+4\left[k_{z}^{2}-E_{B}\left(\gamma E_{B}+\Delta \right)\right]}, \end{array}$$

and

(5) $$\begin{array}{ccc} A_{n,\lambda } & = & \frac{1}{\sqrt{1+b_{n,\lambda }b_{n,\lambda }^{*}}}\\ B_{n,\lambda } & = & \frac{b_{n,\lambda }}{\sqrt{1+b_{n,\lambda }b_{n,\lambda }^{*}}}, \end{array}$$

where λ=±1, $E_{B}=\frac{2n+1}{l_{B}^{2}}$ and $b_{n}=-\frac{l_{B}^{2}\left(\Delta +E_{n,\lambda ,k_{z}}\right)+\gamma E_{B}}{il_{B}^{2}k_{z}}$.

We get $E_{n,\lambda }=\frac{1}{2}\left[E_{B}\left(1+\gamma \right)+\Delta \right]\pm \frac{1}{2}\left|E_{B}\left(1-\gamma \right)-\Delta \right|$ at $k_{z}=0$. $E_{n,+(-)}=\gamma E_{B}+\Delta$ and $E_{n,-(+)}=E_{B}$, with $B<\left(>\right)\frac{\Delta }{e(2n+1)(1-\gamma )}$. The characteristics of LLs dependent on magnetic field *B* at $k_{z}=0$ are displayed in Figure 1c. We define the positive (negative) branch is represented by red (blue) line. For LLs, a specific LL is represented by the *n*, and the maximum value about LLs uses the sign the *N*. We find that LLs not only have the phenomenon of slope changing, but the most interesting feature is asymmetry. Before the slope changing, the spans of the positive and negative branches of LL are 2γeB and 2eB, respectively. When the slopes of the positive and negative branches are interchanged, the spans of LLs will also change. When *B* is within a certain range, the maximum energy of the negative branch is less than or equal to the energy of the positive branch zero LL, so that the negative branch and the positive branch are not mixed. When *B* is outside this range, the positive and negative branches are coupled. In this case, the LLs dispersion along $k_{z}$ is presented fixed *B* shown in Figure 1b. We find that, when the Fermi level falls in the mixed LLs, both the conduction band and the valence band pass through the Fermi level. These rich characteristics of LLs make the type-II NLS have interesting magneto-optical conductivity.

The magneto-optical conductivity can be obtained by the Kubo formula, that is:

(6) $$\begin{array}{ccc} \sigma_{\alpha \beta } & = & -\frac{ie^{2}}{2\pi l_{B}^{2}}\sum_{nn^{{}^{\prime }}}\sum_{\lambda \lambda^{{}^{\prime }}}\int \frac{dk_{z}}{2\pi }\frac{f\left(E_{n,\lambda }\right)-f\left(E_{n^{{}^{\prime }},\lambda^{{}^{\prime }}}\right)}{E_{n,\lambda }-E_{n^{{}^{\prime }},\lambda^{{}^{\prime }}}}\\ & \times & \frac{\langle \Psi_{n,\lambda ,k_{z}}|j_{\alpha }|\Psi_{n^{{}^{\prime }},\lambda^{{}^{\prime }},k_{z}}\rangle \langle \Psi_{n^{\prime },\lambda^{\prime },k_{z}}|j_{\beta }|\Psi_{n,\lambda ,k_{z}}\rangle }{\omega +E_{n,\lambda }-E_{n^{{}^{\prime }},\lambda^{{}^{\prime }}}+i\Gamma }, \end{array}$$

where the Fermi Dirac distribution function is $f\left(x\right)=1/\left(1+e^{(x-\mu )/T}\right)$, with the chemical potential μ and the temperature *T*. The Γ denotes impurity scattering rate. The current operator is expressed in $j_{\alpha }=\frac{\partial H_{\alpha }}{\partial k_{\alpha }}$.

With

(7) $$\begin{array}{c} \langle \Psi_{n,\lambda ,k_{z}}|j_{x}|\Psi_{n^{{}^{\prime }},\lambda^{{}^{\prime }},k_{z}}\rangle \langle \Psi_{n^{\prime },\lambda^{\prime },k_{z}}|j_{x}|\Psi_{n,\lambda ,k_{z}}\rangle =\frac{2n{\left(1+\gamma b_{n,\lambda }^{*}b_{n-1,\lambda^{\prime }}\right)}^{2}}{l_{B}^{2}\left(1+b_{n,\lambda }b_{n,\lambda }^{*}\right)\left(1+b_{n-1,\lambda^{\prime }}b_{n-1,\lambda^{\prime }}^{*}\right)}, \end{array}$$

(8) $$\begin{array}{c} \langle \Psi_{n,\lambda ,k_{z}}|j_{x}|\Psi_{n^{{}^{\prime }},\lambda^{{}^{\prime }},k_{z}}\rangle \langle \Psi_{n^{\prime },\lambda^{\prime },k_{z}}|j_{y}|\Psi_{n,\lambda ,k_{z}}\rangle =\frac{2in{\left(1+\gamma b_{n,\lambda }^{*}b_{n-1,\lambda^{\prime }}\right)}^{2}}{l_{B}^{2}\left(1+b_{n,\lambda }b_{n,\lambda }^{*}\right)\left(1+b_{n-1,\lambda^{\prime }}b_{n-1,\lambda^{\prime }}^{*}\right)}, \end{array}$$

the magneto-optical conductivity Re($\sigma_{xx}$) and Im($\sigma_{xy}$) are achieved by

(9) Re(σxx)n=−e22πlB2∑n∑λλ′∫dkz2πf(En,λ)−f(En−1,λ′)En,λ−En−1,λ′×ηn,λ;kzn−1,λ′×[Γ(ω+En,λ−En−1,λ′)2+Γ2+Γ(ω−En,λ+En−1,λ′)2+Γ2],

(10) Im(σxy)n=−e22πlB2∑n∑λλ′∫dkz2πf(En,λ)−f(En−1,λ′)En,λ−En−1,λ′×ηn,λ;kzn−1,λ′×[Γ(ω+En,λ−En−1,λ′)2+Γ2−Γ(ω−En,λ+En−1,λ′)2+Γ2],

where $\eta_{n,\lambda ;k_{z}}^{n-1,\lambda^{\prime }}=\frac{2n{\left(1+\gamma b_{n,\lambda }^{*}b_{n-1,\lambda^{\prime }}\right)}^{2}}{l_{B}^{2}\left(1+b_{n,\lambda }b_{n,\lambda }^{*}\right)\left(1+b_{n-1,\lambda^{\prime }}b_{n-1,\lambda^{\prime }}^{*}\right)}$. The Re($\sigma_{xx}$) and Im($\sigma_{xy}$) are real part of longitudinal and imaginary part of Hall conductivity, respectively.

The positions of the transition peaks in different *B* ranges are different due to the asymmetric LLs. For $B<\frac{\Delta }{e(2n+1)(1-\gamma )}$, the positions of the peaks are:

(11) $$\begin{array}{c} \omega =\left\{\begin{array}{cccc} 2\gamma eB, & \left(n-1\right) & \to & (n),\\ 2eB, & -(n) & \to & -(n-1),\\ \Delta +2eBn(\gamma -1)+eB(\gamma +1), & -(n-1) & \to & (n),\\ \Delta +2eBn(\gamma -1)-eB(\gamma +1), & -(n) & \to & (n-1). \end{array}\right. \end{array}$$

For $B>\frac{\Delta }{e(2n+1)(1-\gamma )}$, the positions of the peaks are:

(12) $$\begin{array}{c} \omega =\left\{\begin{array}{cccc} 2eB, & \left(n-1\right) & \to & (n),\\ 2\gamma eB, & -(n) & \to & -(n-1),\\ 2eBn(1-\gamma )+eB(1+\gamma )-\Delta , & -(n-1) & \to & (n),\\ 2eBn(1-\gamma )-eB(1+\gamma )-\Delta , & -(n) & \to & (n-1). \end{array}\right. \end{array}$$

## 3. Magneto-Optical Response

**Figure 2:**
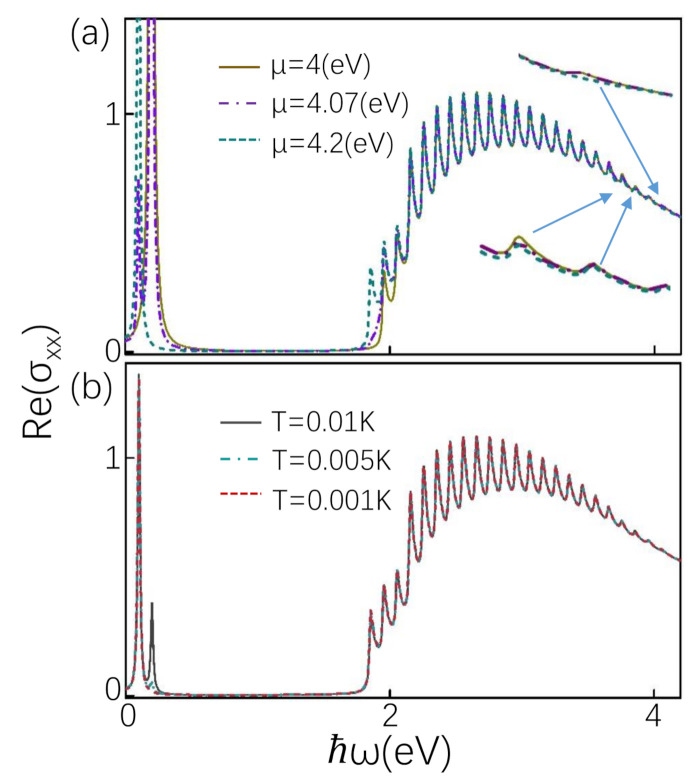
The real part of the longitudinal conductivity as a function of frequency (in units of $\frac{e^{2}}{2\pi l_{B}}$). (**a**) is plotted in several values of μ with B=0.1 T and T=0.01 K. (**b**) The effect of temperature on the optical conductivity is plotted in several values of *T* with B=0.1 T and μ=4.12 eV. The parameters N=20, Δ=4 eV, γ=0.5, Γ=0.01 eV and e=1.

The longitudinal conductivity $\sigma_{xx}$ has significant characteristics affected by asymmetrical LLs. The energy required to excite electronic transition from −(n−1)th to *n*th is different from the energy required from −nth to (n−1)th. However, because the slopes of the positive and negative branches are different, the energy required from −(n−1)th to *n*th and $-n^{\prime }$th to $\left(n^{\prime }-1\right)$th are the same in some cases. According to the positions of the interband peaks in Equations (11) and (12), we can calculate that, at $|n-n^{\prime }|$=$\frac{1+\gamma }{1-\gamma }$, the transition from −(n−1)th to *n*th requires the same energy as the transition from $-n^{\prime }$th to $\left(n^{\prime }-1\right)$th. With γ=0.5, the difference is $|n-n^{\prime }|=3$. Which is to say, the first three peaks only come from −nth to (n−1)th transition, and the last three peaks only are from $-(n^{\prime }-1)$th to $n^{\prime }$th transition. The other oscillation peaks are composed of −nth to (n−1)th transitions and $-(n^{\prime }-1)$th to $n^{\prime }$ transitions. Therefore, the amplitudes of the first three peaks and the last three peaks in Figure 2a are lower. There may be two low amplitude peaks, which are studied detailedly in the following.

Figure 2a shows the real part of the longitudinal conductivity at $B<\frac{\Delta }{e(2n+1-\gamma )}$. From the position of the peak Equation (11), we can see that the position of the peak moves to the low frequency with the increase of *n*. We study the influence of the chemical potential between different LLs on the oscillation peaks. When the chemical potential is above the negative branch and below the zero energy level of the positive branch, seen as μ between n=−19 and n=0 in Figure 1d, taking μ=4 eV, only the amplitude of the first two interband peaks of the optical conductivity is lower, as shown in Figure 2a. Because the chemical potential is below the n=−20 of the negative branch, the interband transition from n=−20 to n=19 is Pauli blocked. The amplitude of the first peak is lower than the original peak because the transition from n=−19 to n=18 is decorated and redistributed to the intraband transition. The position of intraband peak is ω=2eB. When the chemical potential is between n=1 and n=2 of the positive branch, i.e., μ=4.2 eV, an intraband peak appears at ω=2γeB. Low frequency peaks are not affected, and the corresponding high frequency peak disappears, as shown in Figure 2b.

There are two intraband transitions when the chemical potential falls between n=0 (positive branch) and n=−20 (negative branch), taking μ=4.07 eV. This is different from a typical one intraband transition as the μ is between the LLs. According to the transition rules, one transition occurs between the positive branches, i.e., from n=0 to n=1 and the other between the negative branches, i.e., from n=−19 to n=−20, which can be seen from the two close black arrows in Figure 1d. The positions of two intraband peaks are ω=2γeB and ω=2eB, respectively, shown in Figure 2a. When μ is between n=−20 and n=1, taking μ=4.12 eV, optical transitions from n=0 to n=1 and from n=−19 to n=−20 are possible. Because we consider optical transitions at a finite temperature, the transition from n=−19 to n=−20 is possible. This optical transition disappears when the temperature approaches zero. With the decrease of temperature, the possibility of this optical transition becomes smaller and smaller, and finally disappears, which can be clearly observed in Figure 2b.

Furthermore, the most prominent feature is that the oscillation peaks have a non-linear background shown in Figure 2a. This can be explained according to the formula Equation (9), the weights of the peaks are $|Re\left(\sigma_{xx}\right)|\propto \frac{n\gamma^{2}B}{\omega }$. A more physical explanation is that tilted cone can make the background no longer linear [50]. This is different from the typical background of longitudinal conductivity of type-I NLS, which is linear at first and then flat [27,28]. The effect of the tilt term on the background has also been studied in type-II weyl semimetal, which makes its background no longer linear compared with type-I weyl semimetal [50].

**Figure 3:**
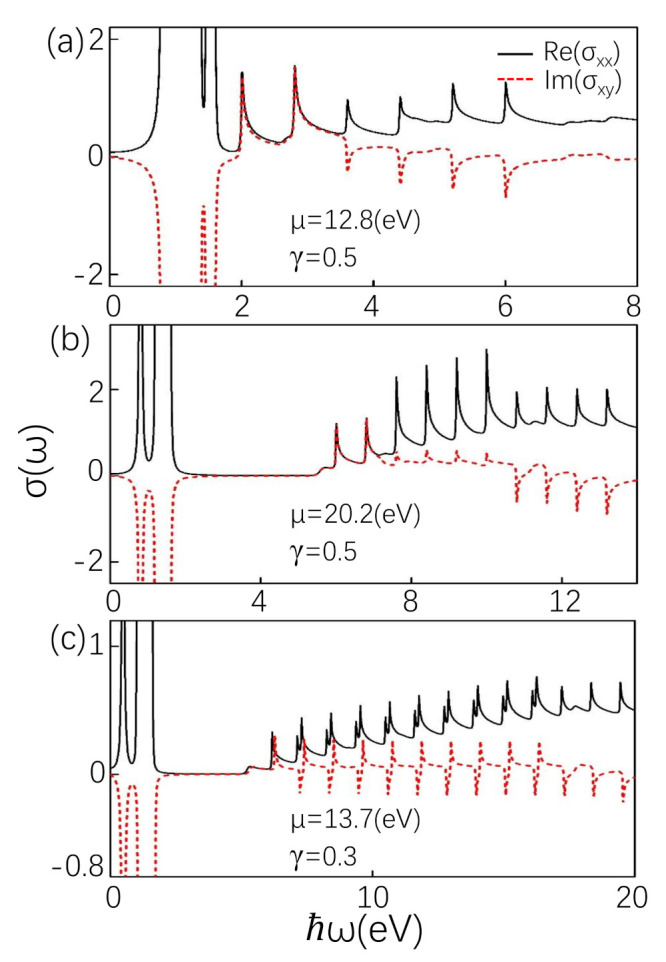
(**a**,**b**) The $Re(\sigma_{xx})$ (solid curve) (in units of $\frac{e^{2}}{2\pi l_{B}}$) compared with $Im(\sigma_{xy})$ (dotted curve) (in units of $\frac{e^{2}}{2\pi l_{B}}$) are plotted for μ=12.8 eV and μ=20.2 eV. The effect of tilt term on the optical conductivity in units of $\frac{e^{2}}{2\pi l_{B}}$. (**c**) $Re(\sigma_{xx})$ and $Im(\sigma_{xy})$ are plotted in γ=0.3 and μ=13.7 eV. The parameters B=0.8 T, and T=0.01 K. Other parameters are the same as in Figure 2.

When $B>\frac{\Delta }{e(2n+1)(1-\gamma )}$, the slopes of the positive and negative *n*th LL are interchanged. At this time, the oscillation peaks are tailless. When the chemical potential lies between the mixed LL, taking μ=12.8 eV, there are also two intraband transitions and several rather than a series of interband transitions shown in Figure 3a. The two intraband transitions are from n=7 to n=8 of the positive branch and from n=−10 to n=−11 of the negative branch near the chemical potential, seen in Figure 1e. The locations of the oscillation peaks can be seen from Equation (12). Because some LLs of positive branches are below the chemical potential, the interband transition from negative branches to these positive branches disappears. In the same way, because some LLs of the negative branch are above the chemical potential, the transition from these LLs to LLs of the positive branch disappears. Hence, for coupled LLs, the peaks are tailless, which is obviously different from the typical characteristics of peaks with a long tail. When μ=12.8 eV, it is above n=7 and below n=−11. Because *n* can only be changed by 1 according to the optical selection rule, the number of LLs that can transition between interband is limited, that is, the LLs of the negative branch are −10≤n≤−7, and the LLs of the positive branch are 8≤n≤11. Therefore, the number of transitions that can occur is 6, and the number of corresponding peaks is 6, shown in Figure 3a. When moving μ gradually leaves the mixed LLs, the number of peaks increases, but it is still tailless. Longitudinal absorption peaks can be clearly seen from Figure 3b, taking μ=20.2 eV. Tailless optical conductivity also exists in coupled LLs region Δ/e(2N+1−γ)<B<Δ/e(2N+1)(1−γ). The reason is that the mixed LLs depending on *B* have a unusual dispersion relation with $k_{z}$. In a finite $k_{z}$, both positive and negative branches pass through the Fermi level, which is similar to that of type-II Weyl Semimetal [51].

The influence of asymmetry energy level of positive and negative branches is even more striking for the Hall conductivity than for longitudinal conductivity. We find that absorption peak of Hall conductivity (Im$\left(\sigma_{xy}\right)$) has some positive oscillations within a certain frequency and then negative at high frequencies displayed in Figure 3b. Physically, the Hall conductivity is derived from the contribution of the four transitions: (i) the interband transition from particle-branch LLs −(n−1)th to hole-branch LLs *n*, (ii) the interband transition from particle-branch LLs −nth to hole-branch LLs (n−1), (iii) the intraband transition in hole-branch LLs from (n−1) to *n*, and (iv) the intraband transition in particle-branch LLs from (n−1) to *n*. On the basis of Equation (10), the contributions of (i) and (ii) are opposite sign, and contributions of (iii) and (iv) are same in sign. Since the LLs below the chemical potential are unoccupied states, the transition of the particle and hole branchs from *n* to (n−1) does not occur. Therefore, in Figure 3a,b, there is two intraband transition at low frequency, which is opposite to the sign of Re$\left(\sigma_{xx}\right)$. Moreover, asymmetric particle-hole and coupled LLs make the interband peaks in Im$\left(\sigma_{xy}\right)$ nonzero. The first three interband peaks of Hall conductivity are positive and the last three interband peaks are negative, according to (i) and (ii), shown in Figure 3a,b. Due to the above-mentioned, the positions of the intreband transition peaks from −(n−1)th to *n*th and $-n^{\prime }$th to $\left(n^{\prime }-1\right)$th are the same with $n^{\prime }-n$ = $\frac{1+\gamma }{1-\gamma }$. However, the weights of the intreband transition peaks are different with $|Im\left(\sigma_{xy}\right)|\propto \frac{n\gamma^{2}B}{\omega }$ according to Equation (10). Therefore, some weak positive oscillations are found in Figure 3b.

The asymmetry of particle and hole branches is mainly affected by tilt term γ. When the value of γ cannot satisfy that $\frac{1+\gamma }{1-\gamma }$ is a positive integer, the characteristics of Hall conductivity and longitudinal conductivity are obviously different from the above. When the difference between *n* and $n^{\prime }$ is close to $\frac{1+\gamma }{1-\gamma }$, the peaks from −(n−1)th to *n*th and $-n^{\prime }$th to $\left(n^{\prime }-1\right)$th are close, shown in Figure 3c. Moreover, the oscillation intensity from −(n−1)th to *n*th is lower than that from $-n^{\prime }$th to $\left(n^{\prime }-1\right)$th complying with $|Im\left(\sigma_{xy}\right)|\propto \frac{n\gamma^{2}B}{\omega }$. According to the above theoretical analysis, Hall conductivity shows two adjacent peaks with opposite signs, as we can see from Figure 3c.

**Figure 4:**
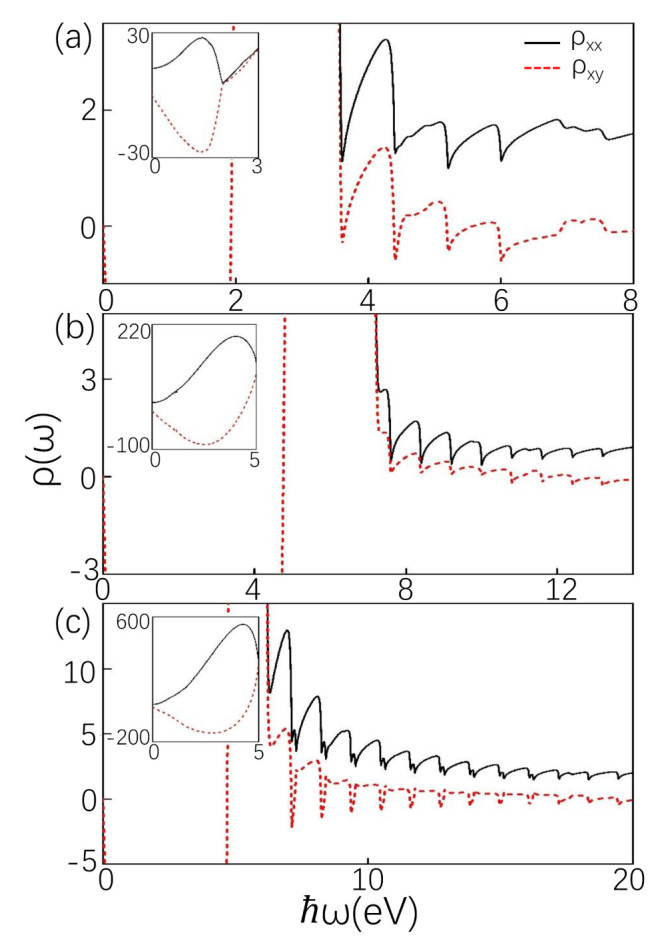
Resistivity in units of $2\pi l_{B}$. (**a**–**c**) corresponds to (**a**–**c**) in Figure 3. The parameters are the same as in Figure 3.

Experimentally, elements of the longitudinal resistivity tensor $\rho_{xx}$ and Hall resistivity tensor $\rho_{xy}$ can be obtained with formulas Equations (9) and (10) via expressions $\rho_{xx}=\sigma_{xx}/S$ and $\rho_{xy}=\sigma_{xy}/S$, where $S=\sigma_{xx}\sigma_{yy}-\sigma_{xy}\sigma_{yx}$ [52,53,54]. The component $\sigma_{yy}=\sigma_{xx}$ and $\sigma_{yx}=-\sigma_{xy}$. Thus, $\rho_{xx}=\sigma_{xx}/\left[\sigma_{xx}^{2}+\sigma_{xy}^{2}\right]$ and $\rho_{xy}=\sigma_{xy}/\left[\sigma_{xx}^{2}+\sigma_{xy}^{2}\right]$. Figure 4 presents longitudinal resistivity $\rho_{xx}$ and Hall resistivity $\rho_{xy}$ as a function of photon energy for different chemical potentials and tilt terms, fixed B=0.8 T, T=0.01 K. Intraband transitions at low frequencies have a large resistivity displayed in the enlarged image in Figure 4. The oscillating peak related to interband transition of the resistivity is just the opposite of the oscillating peak of the conductivity.

**Figure 5:**
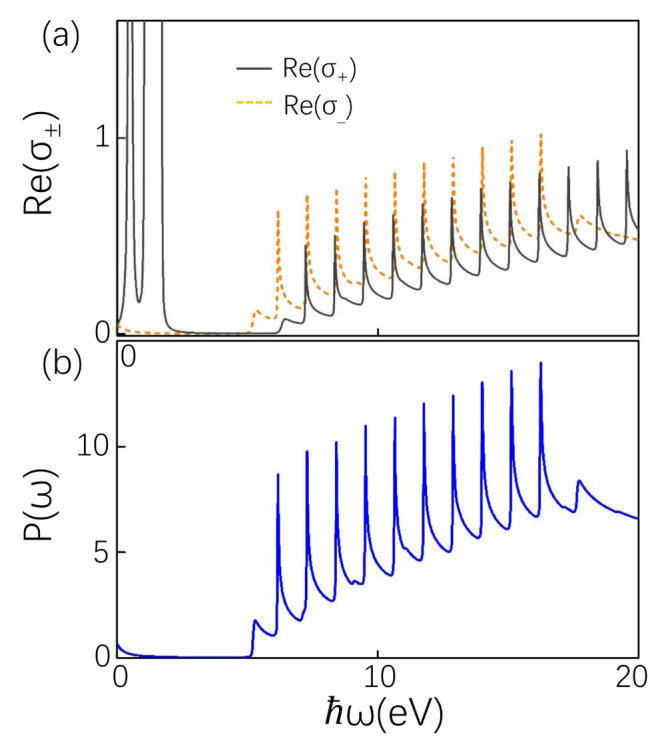
(**a**) The absorption part of the optical conductivity (in units of $\frac{e^{2}}{2\pi l_{B}}$) for circularly polarized light. The parameters are the same as in Figure 3c. (**b**) Power spectrum (in units of $\frac{e^{2}}{2\pi l_{B}}$) versus energy. The parameters are the same as (**a**).

Then, we study the circularly polarized light in type-II NLS that can be detected in experimentally, just as the Faraday and Kerr effects, which is quantified as $\sigma_{\pm }=\sigma_{xx}\pm i\sigma_{xy}$. The left- and right-handed polarized lights are represented by $\sigma_{-}$ and $\sigma_{+}$, respectively. The absorptive part of the conductivity is Re$\left(\sigma_{\pm }\right)$ = Re$\left(\sigma_{xx}\right)$ ∓ Im$\left(\sigma_{xy}\right)$. With Equations (9) and (10), the absorptive parts for left- and right-handed polarized lights are plotted as a function of ω in Figure 5a. In graphene, Weyl semimetal, and other materials, the most typical characteristic of circularly polarized light is that the amplitudes of the peaks for left-handed (right-handed) circularly polarized light at low frequency are twice that of optical conductivity (disappear), and other peaks are consistent with the longitudinal conductivity [41,55,56,57,58]. For type-II NLS, there are several important differences. The closed peaks are absent in left- and right-handed polarized lights compared with optical conductivity. In addition, not only the positions of $\sigma_{-}$ and $\sigma_{+}$ peaks are different, but also the amplitudes of $\sigma_{-}$ peaks are stronger than that of $\sigma_{+}$. The $\sigma_{-}$ ($\sigma_{+}$) peaks are from the transition between −n and (n−1) (between −(n−1) and *n*). And the doubling and disappearance of peaks not only occur at low frequencies, but similar situations also occur in high frequency intreband transitions. These interesting phenomena are attributed to asymmetry of particle and hole branches.

The difference between $\sigma_{-}$ and $\sigma_{+}$ can also be displayed in the power absorption spectrum given by [59]

(13) $$\begin{array}{ccc} P(\omega ) & = & \frac{E}{2}\left[\sigma_{xx}\left(\omega \right)+\sigma_{yy}\left(\omega \right)-i\sigma_{yx}\left(\omega \right)+i\sigma_{xy}\left(\omega \right)\right]. \end{array}$$

The spectrum P(ω) as a function of energy is shown in Figure 5b. Since the negative part of $\sigma_{xy}$ is the positive part of $\sigma_{xx}$ (see Equations (9) and (10)), its peak is basically the same as the peak of longitudinal optical conductivity, instead of alternating positive and negative.

For further understand Hall conductivity of type-II NLS. The magnetic oscillation is studied in $\sigma_{xy}$, which can be obtained from the standard formula in the linear response theory [60,61,62].

(14) $$\begin{array}{ccc} \sigma_{xy} & = & -\frac{i\hbar e^{2}}{V}\sum_{\zeta \neq \zeta^{\prime }}f_{\zeta }\left(1-f_{\zeta^{\prime }}\right)\langle \zeta |j_{x}|\zeta^{\prime }\rangle \langle \zeta^{\prime }|j_{y}|\zeta \rangle \frac{1-e^{\beta (E_{\zeta }-E_{\zeta^{\prime }})}}{{\left(E_{\zeta }-E_{\zeta^{\prime }}\right)}^{2}}. \end{array}$$

Due to $f_{\zeta }\left(1-f_{\zeta^{\prime }}\right)\left(1-e^{\beta (E_{\zeta }-E_{\zeta^{\prime }})}\right)$ = $f_{\zeta^{\prime }}\left(1-f_{\zeta }\right)$, one can arrive

(15) $$\begin{array}{ccc} \sigma_{xy} & = & -\frac{i\hbar e^{2}}{V}\sum_{\zeta \neq \zeta^{\prime }}\left(f_{\zeta }-f_{\zeta^{\prime }}\right)\frac{\langle \zeta |j_{x}|\zeta^{\prime }\rangle \langle \zeta^{\prime }|j_{y}|\zeta \rangle }{{\left(E_{\zeta }-E_{\zeta^{\prime }}\right)}^{2}}, \end{array}$$

where *V* is volume, and $|\zeta \rangle \equiv \Psi_{n,\lambda ,k_{z}}$,

(16) $$\begin{array}{c} \frac{1}{V}\sum_{k}=\frac{1}{{\left(2\pi \right)}^{3}}\int dk_{z}. \end{array}$$

According to Equation (8), we know

(17) $$\begin{array}{c} \langle \zeta |j_{x}|\zeta^{\prime }\rangle \langle \zeta^{\prime }|j_{y}|\zeta \rangle =i\eta_{n,\lambda ;k_{z}}^{n-1,\lambda^{\prime }}. \end{array}$$

Substituting Equations (16) and (17) into Equation (15), we obtain the Hall conductivity as

(18) $$\begin{array}{ccc} \sigma_{xy} & = & \frac{\hbar e^{2}}{{\left(2\pi \right)}^{3}}\int dk_{z}\sum_{\zeta \neq \zeta^{\prime }}\left(\eta_{n,\lambda ;k_{z}}^{n-1,\lambda^{\prime }}\right)\frac{f_{\zeta }-f_{\zeta^{\prime }}}{{\left(E_{\zeta }-E_{\zeta^{\prime }}\right)}^{2}}. \end{array}$$

**Figure 6:**
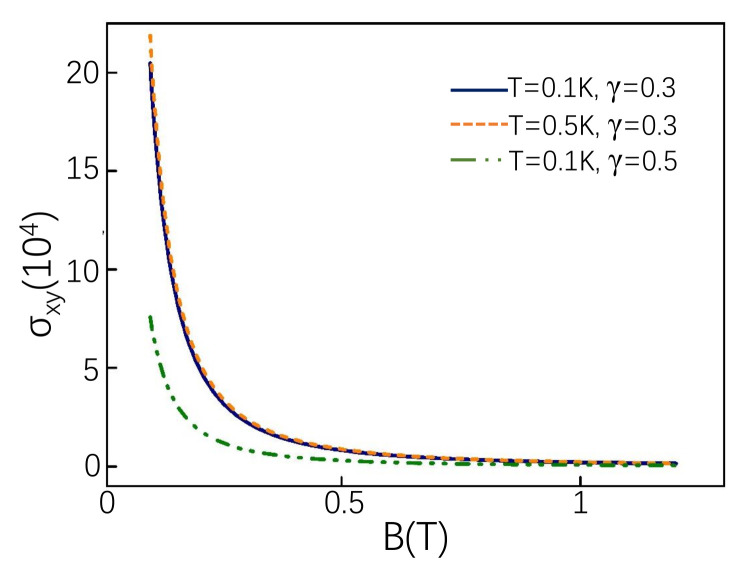
Hall conductivity(in units of $\frac{e^{2}}{{\left(2\pi \right)}^{2}}$) as function of magnetic field at different temperatures and tilt terms. The parameters μ=20.2 eV for γ=0.5 and μ=13.7 eV for γ=0.3. Similar to the above, we take e=1 and ℏ=1.

The amplitude of oscillations is hardly affected by temperature, but it is sensitive to the tilt term seen from Figure 6. The amplitude of oscillations is reduced considerably at relatively large tilt term. As *B* increases, the oscillation is greatly reduced. At the same time, the influence of the tilt term is gradually weakened and no longer have an impact in the end.

## 4. Discussion and Summary

In this work, we study the magneto-optical transport properties of type-II NLS. The characteristics of the magneto-optical conductivity are closely related to the tilted linear dispersion. Two Dirac cones with different slop make the conduction band and valence band asymmetric. When the magnetic field is perpendicular to the plane of the ring, the positive and negative branches of LLs are also asymmetric. This means that the energy required for the intreband transition from −(n−1) in particle branch to *n* in hole branch is not the same as from −n to (n−1). However, the peaks for interband transitions are the same in position or very close, each carrying a different optical spectral weight, when $|n-n^{\prime }|=\left(\approx \right)\frac{1+\gamma }{1-\gamma }$. Additionally, the interband transitions depend on relative magnitude of *B* to $\frac{\Delta }{e(1-\gamma )}$. The oscillation background of the peak is curved affected by tilt term γ. Moreover, the LLs of the particle and hole branches are partially coupled when $B\geq \frac{\Delta }{e(2N+1-\gamma )}$. In this case, the peak does not have a long tail because the particle and hole LLs pass through the Fermi level in a finite $k_{z}$. Hall conductivity is affected by tilt term γ, which shows two adjacent peaks with opposite signs, or negative peaks are at higher frequency relative to positive peaks. In addition, we find the magnetic oscillation of Hall conductivity is insensitive to temperature and sensitive to γ. The absorption peaks of circularly polarized light are also studied. The peaks of the left and right polarized light are not only different in position, but the oscillation intensity is also different. In particular, the unique circularly polarized light and resistivity of type-II NLS can be used to detect whether the material is type-II NLS in the experiment.

## Data Availability

Not applicable.
